# RDYH58 functional exosomes targeting myofibroblasts loaded with siFKBP10 for inhibition of collagen biosynthesis and secretion of IPF

**DOI:** 10.1016/j.apsb.2025.08.017

**Published:** 2025-08-27

**Authors:** Ranran Yuan, Zhen Mu, Houqian Zhang, Yu Tian, Quanlin Xin, Qingchao Tu, Yan Zhang, Yanqiu Li, Zhiwen Zhang, Yongchao Chu, Aiping Wang, Jingwei Tian, Hongbo Wang, Chong Qiu, Yanan Shi

**Affiliations:** a School of Pharmacy, Key Laboratory of Molecular Pharmacology and Drug Evaluation, Ministry of Education, Collaborative Innovation Center of Advanced Drug Delivery System and Biotech Drugs in Universities of Shandong, Yantai University, Yantai 264005, China; b State Key Laboratory for Quality Ensurance and Sustainable Use of Dao-Di Herbs, Artemisinin Research Center, and Institute of Chinese Materia Medica, China Academy of Chinese Medical Sciences, Beijing 100700, China; c Department of Respiratory and Critical Care Medicine, Yantai Yuhuangding Hospital, Affiliated with the Medical College of Qingdao, Yantai 264200, China; d School of Pharmacy, Fudan University, Shanghai 200437, China

**Keywords:** Idiopathic pulmonary fibrosis, Myofibroblasts, FK506-Binding protein, RDYH58, Exosomes, Drug delivery, Small interfering RNA, Ultrasonic microfluidics method

## Abstract

Idiopathic pulmonary fibrosis (IPF) is a complex interstitial lung disease in which myofibroblasts are the primary effector cells. FK506-binding protein (FKBP10), a procollagen chaperone, is upregulated in IPF and primarily localizes to myofibroblasts. Exosomes have garnered significant attention as novel drug delivery vehicles, particularly when engineered. However, myofibroblasts remain underexplored in terms of engineered exosome-based therapies and associated drug targets. In this study, RDYH58, a peptide that targets myofibroblasts, was conjugated to the exosomal membrane protein Lamp2b to produce RDYH58-linked exosomes (RDYH58-exo). *In vitro* and *in vivo* experiments demonstrated that compared to unmodified exosomes (unm-exo), RDYH58-exo preferentially localized to myofibroblasts. A small interfering RNA targeting FKBP10 (siFKBP10) was loaded into exosomes using ultrasonic microfluidics method, and the antifibrotic effects of RDYH58-exo carrying siFKBP10 (RDYH58-siFKBP10) were assessed both *in vitro* and *in vivo*. The results demonstrated that RDYH58-siFKBP10 effectively silenced FKBP10 gene expression, significantly inhibiting fibroblast activation and extracellular matrix deposition, with superior antifibrotic efficacy compared to unmodified exosome vectors (unm-siFKBP10). RNA-seq analysis confirmed the pivotal regulatory role of FKBP10, providing critical evidence for the development of targeted therapeutic strategies. The RDYH58-siFKBP10 delivery system developed in this study demonstrates remarkable clinical translation potential.

## Graphical abstract

This study utilized exosomes targeting myofibroblasts (RDYH58-exo) loaded with FKBP10-specific small interfering RNA (RDYH58-siFKBP10) to assess their anti-fibrotic effects both *in vitro* and *in vivo*.

**Figure:**
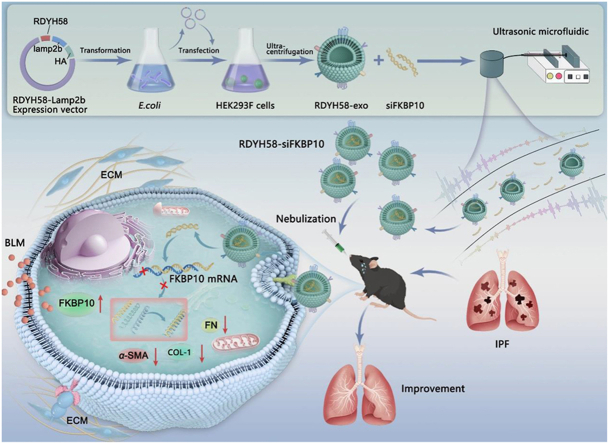

## 1 Introduction

Exosomes are small vesicles with a diameter of 30‒150 nm secreted by all cell types and represent the smallest subtype of extracellular vesicles. Owing to their origin and small size, exosomes are less likely to be phagocytosed by macrophages and lysosomes^1^, making them ideal vehicles for the delivery of small nucleic acids^2^^,^^3^, peptides^4^, and small-molecule compounds^5^^,^^6^. Several methods have been developed to facilitate the loading of small nucleic acids into exosomes, including electroporation^7^, ^8^, ^9^, sonication^10^^,^^11^, and modified calcium chloride-mediated transfection methods^12^. However, these methods typically have a relatively low loading efficiency^13^. Despite these limitations, targeted delivery can enhance local concentrations of therapeutic agents and reduce their potential side effects. Exosomes, as naturally derived carriers, offer a unique advantage because their surface, enriched with membrane proteins, allows for convenient modification. Currently, exosome modification methods can be broadly categorized into genetic engineering, chemical modification, and membrane fusion^14^, with the latter two inevitably increasing the drug toxicity of the delivery vehicle. Genetic engineering involves linking the gene sequence of a specific protein or peptide to that of an exosomal membrane protein, thereby effectively anchoring the protein or peptide to the exosome surface.

Idiopathic pulmonary fibrosis (IPF) is a progressive restrictive lung disease^15^, characterized by complex mechanisms, signaling pathways, and cellular interactions. Patients diagnosed with acute exacerbations of IPF have a median survival of approximately 3‒5 years^16^, with particularly high morbidity and mortality rates in Europe and North America^17^. IPF is commonly associated with abnormal epithelial cell activation, repeated injury, and impaired repair of lung tissue. The interaction between abnormal epithelial cells and immune cells leads to the activation of fibroblasts, excessive secretion of transforming growth factor-*β* (TGF-*β*), and the deposition of extracellular matrix (ECM), such as collagen I (COL-1), impairing gas exchange between alveoli^18^. During IPF progression, myofibroblasts are the primary effector cells^19^, predominantly arising from activated lung fibroblasts, and are characterized by the secretion of alpha-smooth muscle actin (*α*-SMA)^20^. FK506-binding protein (FKBP10) was initially discovered in mouse fibroblasts, localized in the rough endoplasmic reticulum, and is involved in procollagen biosynthesis^21^. FKBP10 exhibits peptidylprolyl *cis/trans* isomerase activity, which is required for the isomerization of proline to transproline during the assembly of linear collagen chains into a collagen triple helix^22^. FKBP10 directly interacts with COL-1 within cells, and its expression is developmentally regulated in postnatal bones and ligaments but reactivated in injured tissues. Mutations in FKBP10 can lead to recessive osteogenesis imperfecta and Bruck syndrome^23^^,^^24^. FKBP10 has been identified as a therapeutic target for IPF because of its role in collagen regulation. The downregulation of FKBP10 expression can reduce collagen and fibronectin (FN) production in lung fibroblasts and inhibit the migration of myofibroblasts^25^. Currently, no small-molecule inhibitors targeting FKBP10 are available, and the lack of understanding of the three-dimensional structure of FKBP10 hinders the development of structure-based inhibitors^26^. The approval of the first small interfering RNA (siRNA) drug has paved the way for the use of siRNAs in the treatment of other diseases^27^. The transcription sequence of FKBP10 can be used to design an FKBP10-specific siRNA (siFKBP10). However, the physiological characteristics of the lungs and the structural features of siRNAs make it challenging to deliver siRNA drugs to specific cells in the lungs. Given the direct exposure of lungs to the external environment, inhaled siRNA-based therapies hold great promise. An efficient inhalation delivery system will not only reduce the required dosage but also minimize the side effects caused by metabolism^28^.

Recent advancements in the treatment of IPF have led to the development of engineered exosomes as a novel drug delivery platform. These systems achieve targeted drug delivery to lung tissues or specific cell types, thereby enhancing therapeutic efficacy. However, myofibroblasts, which are key players in IPF pathogenesis, remain underexplored in terms of engineered exosome-based therapies and associated drug targets. In this study, RDYH58 is a peptide designed to specifically target myofibroblasts^29^. By engineering a fusion protein comprising RDYH58 and Lamp2b, exosomes were isolated and conjugated with RDYH58 (RDYH58-exo). FKBP10, which is primarily localized within myofibroblasts, serves as a therapeutic target. Using ultrasonic microfluidics, siFKBP10 was encapsulated into these exosomes. The uptake of RDYH58-exo by myofibroblasts was evaluated both *in vitro* and *in vivo*. Additionally, the antifibrotic effects of RDYH58-exo loaded with siFKBP10 (RDYH58-siFKBP10) were assessed in bleomycin (BLM)-induced cellular models and IPF mouse models. The results indicate that the combination of RDYH58-exo and siFKBP10 represents a promising approach for ameliorating IPF, demonstrating potential for targeted therapy against myofibroblasts, a key cell type involved in disease progression. This method offers a novel strategy for improving the specificity and effectiveness of IPF treatments.

## 2 Materials and methods

### 2.1 Plasmids

The cDNA sequence of the RDYH58 peptide was predicted using the EMBOSS Backtranseq tool, yielding the sequence: AGGGACTACCACCCCAGGGACCACACCGCCACCTGGGGCGGCTGC. Primers were designed at both ends of the RVG sequence on the plasmid vector (GNSTM-RVG-Lamp2b-HA) to facilitate circular plasmid amplification. Homologous recombination was then performed to replace the RVG peptide in the vector with the RDYH58 peptide (GNSTM-RDYH58-Lamp2b-HA). The recombinant plasmid was transformed into DH5*α* chemically competent cells (Vazyme, C502, Nanjing, China) and cultured overnight at 37 °C in liquid Luria-Bertani medium. Plasmids were extracted using the plasmid extraction kit (Vazyme, DP117). The plasmid expressing RDYH58-Lamp2b-HA was transfected into HEK293F cells, and the exosomes expressing RDYH58-Lamp2b-HA were isolated.

### 2.2 Preparation of exosomes

HEK293F cells (Thermo Fisher Scientific, A14527) were cultured in a chemically defined, serum-free, and protein-free Expi293™ Expression Medium (Thermo Fisher Scientific, A1435103). According to the manufacturer's instructions, transfection was performed when cell density reached 1.5 × 10^6^‒2.5 × 10^6^ cells/mL. The transfection reagent (Beyotime, C0518, Shanghai, China) was mixed with the GNSTM-RDYH58-Lamp2b-HA plasmid and incubated for 15 min at room temperature before being added to the culture medium. The cells were then cultured for an additional 48‒72 h.

HEK293F cells were cultured in a shaker incubator (Shanghai Zhichu Instruments Co., Ltd., ZCZY-AS8E, Shanghai, China) at 37 °C with 5% CO_2_. When the cell density reached 6 × 10^6^‒8 × 10^6^ cells/mL, exosomes were isolated using differential ultracentrifugation. Briefly, differential centrifugation was used to remove cells and cell debris, followed by filtration through a 0.22 μm filter. Exosomes were then collected by ultracentrifugation at 100,000 *×* *g* using an ultracentrifuge (Thermo Fisher Scientific, Sorvall WX100+) and resuspended in phosphate-buffered saline (PBS). Both RDYH58-exo and unmodified exosomes (unm-exo) were isolated. The protein concentration of the exosomes was measured using the BCA protein assay.

### 2.3 Transmission electron microscopy

Using tweezers, place the copper grid horizontally and add 4 μL of the sample onto the grid. Allow it to stand for 1 min, then remove the excess sample with filter paper. Next, add 4 μL of phosphotungstic acid to the grid, let it stand for approximately 45 s, and remove the excess phosphotungstic acid with filter paper. After air-drying, observe the morphology and structure of the exosomes using transmission electron microscopy (TEM, JEM-1230, Tokyo, Japan).

### 2.4 Nanoparticle tracking analysis

Dilute the exosomes to an appropriate concentration in PBS and load them into a syringe. Then, place the syringe on an automatic infusion pump and analyze the particle concentration of the exosomes using nanoparticle tracking analysis (Malvern Panalytical, NTA, Great Malvern, UK).

### 2.5 Preparation of siFKBP10-loaded exosomes

Exosomes were loaded with siFKBP10 using ultrasonic microfluidics method. Briefly, exosomes and siRNA were mixed at a mass ratio of 1:1 in 1 mL PBS buffer, and an automatic syringe pump was set at a flow rate of 200 μL/min. The ultrasonic generator was operated at a power of 30 W. Samples were collected 1 min following the start of the injection. The total siRNA content in the samples was quantified using the Quant-iT RiboGreen RNA Assay kit (Invitrogen, R11490, Carlsbad, CA, USA).

### 2.6 Western blotting analysis

Cells and lung tissues were lysed using RIPA lysis buffer (Meilunbio, MA0151, Dalian, China), and total proteins were extracted by centrifugation. Proteins were separated on 10% SDS-PAGE and transferred onto a PVDF membrane (Merck Millipore, ipvh00010, Burlington, VT, USA) using an electrophoresis and transfer system (Bio-Rad, Hercules, CA, USA). The PVDF membrane was incubated overnight at 4 °C with the following primary antibodies: anti-HA (Proteintech, 51064-2-AP, Chicago, IL, USA), anti-CD9 (Abcam, ab236630, Cambridge, UK), anti-CD81 (Abcam, ab109201), anti-TSG101 (Abcam, ab125011), anti-CD63 (Abcam, ab68418), anti-GAPDH (Proteintech, 60004-1-Ig), anti-FKBP10 (Proteintech, 12172-1-AP), anti-*α*-SMA (ABclonal, A17910, Wuhan, China), anti-COL-1 (Cell Signaling, 72026, Danvers, MA, USA), and anti-FN (Abcam, ab2413). Necessary visualization was performed using a chemiluminescence detection system (Analytik, Jena, Jena, Germany).

### 2.7 Cell transfection

According to the manufacturer's instructions, transfection reagent (Beyotime, C0518) was mixed at a specific ratio and allowed to stand for 15 min at room temperature before being added to the cell suspension. The cells were cultured for an additional 48‒72 h.

### 2.8 Cell culture and BLM-induced cell fibrotic model

Human fetal lung fibroblast 1 (HFL-1) cells, obtained from the Chinese Academy of Sciences (Shanghai, China), were cultured in Ham's F-12K medium (Thermo Fisher Scientific, 21127022) supplemented with 10% FBS (Thermo Fisher Scientific, C0235) in a 37 °C, 5% CO_2_ incubator. When the HFL-1 cells reached 80% confluency, the medium was replaced with fresh medium containing BLM (Maokang Biotechnology Co., Ltd., 9041-93-4, Shanghai, China) to induce fibrotic injury in the cells.

### 2.9 DiO-labeled exosomes and cellular uptake

10 mg of DiO dye (Beyotime, C1038) was dissolved in DMSO to prepare a 5 mmol/L stock solution. This stock solution was then diluted in PBS to achieve a 500 μmol/L working solution. For labeling, 4 μL of the DiO working solution was added to each 100 μL of exosome suspension, yielding a final DiO concentration of 20 μmol/L. The mixture was gently sonicated to ensure thorough mixing of the DiO dye with the exosomes. Subsequently, the labeled exosomes were incubated at 37 °C for 30 min. Unbound dye was subsequently removed *via* ultrafiltration.

HFL-1 cells were seeded into 12-well plates at a density of 6 × 10^4^ cells per well and cultured for 24 h. The medium was then replaced with fresh medium containing DiO-labeled exosomes (DiO-unm-exo and DiO-RDYH58-exo). After 2 and 6 h of incubation, the cells were washed three times with PBS, fixed with 500 μL of 4% paraformaldehyde per well for 15 min, and stained with DAPI antifade mounting medium (Beyotime, P0131) to label the nuclei. Cellular uptake was visualized using a laser scanning confocal microscope (Carl Zeiss AG, LSM800, Oberkochen, Germany).

### 2.10 IPF mouse model

All animal experiments were conducted in accordance with the ethical standards and guidelines established by the Yantai University Animal Care and Use Committee. All animal experiments were approved by the Experimental Animal Ethics Committee of the College of Pharmacy. Yantai University (Approval number, YTDX 20180124). Eight-week-old male BALB/c and C57BL/6 mice, weighing 20‒30 g, were obtained from Jinan Pengyue Experimental Animal Breeding Co., Ltd. (Jinan, China). The mice were acclimatized for two weeks in an environment maintained at 50 ± 10% relative humidity, 24 ± 2 °C, with a 12-h light/dark cycle. For the induction of the IPF mouse model, to minimize interference of dark hair with fluorescence detection, BALB/c mice were selected for establishing the IPF mouse model *in vivo* imaging studies. Male BALB/c mice were given 5 mg/kg of BLM *via* intratracheal instillation, while male C57BL/6 mice received 1.5 mg/kg of BLM. The control group mice were administered an equivalent volume of saline.

### 2.11 *In vivo* tracking of unm-exo/RDYH58-exo

The healthy mice and IPF BALB/c mice were administered 6 × 10^8^ DiO-unm-exo or DiO-RDYH58-exo particles *via* intratracheal nebulization. Using a small animal imaging system (PerkinElmer, IVIS Kinetic, Shelton, WA, USA), the distribution of exosomes within the mice was observed at various time points (4 h, Days 1, 3, 5, and 7).

### 2.12 Immunofluorescence staining

HFL-1 cells were seeded in 12-well plates and allowed to adhere. The cells were induced with bleomycin. Different treatments were applied: PBS, RDYH58-exo, RDYH58-exo loaded with control siRNA (RDYH58-siNC), naked siFKBP10, unm-exo loaded with siFKBP10 (unm-siFKBP10), and RDYH58-exo loaded with siFKBP10 (RDYH58-siFKBP10). After 48‒72 h of culture, the cells were fixed with paraformaldehyde, permeabilized with 0.2% Triton X-100, and blocked with QuickBlock™ Blocking Buffer (Beyotime, P0260). Primary antibody incubation was carried out overnight at 4 °C, followed by secondary antibody incubation at room temperature for 2 h. Nuclei were stained with DAPI, and the cells were observed under a laser scanning confocal microscope.

DiO-unm-exo and DiO-RDYH58-exo were administered to the trachea of control and BLM-treated C57BL/6 mice *via* nebulization. After 24 h, lung tissues were collected and fixed in 4% paraformaldehyde for 24 h, and dehydrated, followed by incubation in a 30% sucrose solution for another 24 h. The lungs were then embedded in optimal cutting temperature (OCT) compound and sectioned into 10-μm slices using a cryostat. The sections were blocked for 60 min and incubated overnight at 4 °C with antibodies against Epithelial Cell Adhesion Molecule (Proteintech, CL555-21050), CD68 (Proteintech, CL594-25747), Platelet Endothelial Cell Adhesion Molecule-1 (Proteintech, CL647-65058), and *α*-SMA (Cell Signaling, P62736). Nuclei were stained with DAPI, and the sections were observed under a laser scanning confocal microscope.

### 2.13 Animal grouping and treatment

Day 7 post-BLM exposure, male C57BL/6 mice were randomly divided into six groups: BLM + PBS, BLM + RDYH58-exo, BLM + RDYH58-siNC, BLM + naked siFKBP10, BLM + unm-siFKBP10, and BLM + RDYH58-siFKBP10. The BLM + RDYH58-siFKBP10 group received 50 μL intratracheal aerosol administration at a dose of 1.5 mg/kg body weight, dissolved in sterile PBS. The control group was given an equal volume of PBS alone. Administration was performed every three days for a total of five doses. On Day 22 of the experiment, mice were euthanized by cervical dislocation, and lung tissues were collected for subsequent analysis.

### 2.14 Cell scratch assay

HFL-1 cells were seeded in 12-well plates. Once the cells reached 90% confluency, a scratch was made in the center of the cell monolayer to create a blank area. Floating cells were washed away with PBS, and the remaining cells were cultured in medium. Cell migration was imaged at 0- and 24-h post-scratch using a live cell imaging system (Cytation, BioTek™, VT, USA), and the migration area was analyzed using ImageJ software.

### 2.15 Histology analysis

Lung tissues were fixed in paraformaldehyde, dehydrated with ethanol, and embedded in paraffin. The sections were cut into 4‒6 μm slices using a microtome. Paraffin sections were deparaffinized in xylene, rehydrated through a graded ethanol series, and washed with PBS. Subsequently, the sections were stained with H&E (Beyotime, C0105S), Masson's trichrome (Solarbio, G1346, Beijing, China), and Sirius Red (Solarbio, G1472) and examined under a microscope (OLYMPUS BX53M, Tokyo, Japan).

Immunohistochemistry: Paraffin sections were deparaffinized and rehydrated, followed by antigen retrieval in a solution heated to above 95 °C. The sections were then incubated with blocking solution. Primary antibody incubation was performed overnight at 4 °C. After washing, the sections were incubated with a biotin-labeled secondary antibody at room temperature for 1 h. Subsequently, the sections were incubated with Streptavidin-Biotin Complex working solution for 30 min. Finally, sections were visualized using DAB chromogen.

### 2.16 Hydroxyproline content measurement

Hydroxyproline detection is used as an alternative measure of collagen content to evaluate the extent of fibrosis. Protein is first extracted and its concentration determined. Hydroxyproline content is then measured using a hydroxyproline assay kit (Solarbio, BCO255), with results normalized to the protein concentration of the sample.

### 2.17 Statistical analysis

All statistical analyses were performed using GraphPad Prism 8 software (La Jolla, San Diego, CA, USA). Data are presented as mean ± standard deviation (SD). All values were assumed to follow a normal distribution. For *in vivo* experiments, *n* = 3‒6 biological replicates were used, and *n* = 3‒4 for *in vitro* experiments. Quantitative analysis of fluorescence signals and Western blot bands was carried out using ImageJ software. For comparisons between two groups, a two-tailed Student's *t*-test was used, and for comparisons among three or more groups, one-way analysis of variance (ANOVA) was applied. *P* < 0.05 was considered statistically significant.

## 3 Result

### 3.1 Preparation and characterization of exosomes/exosomes loaded with siRNA

**Figure 1:**
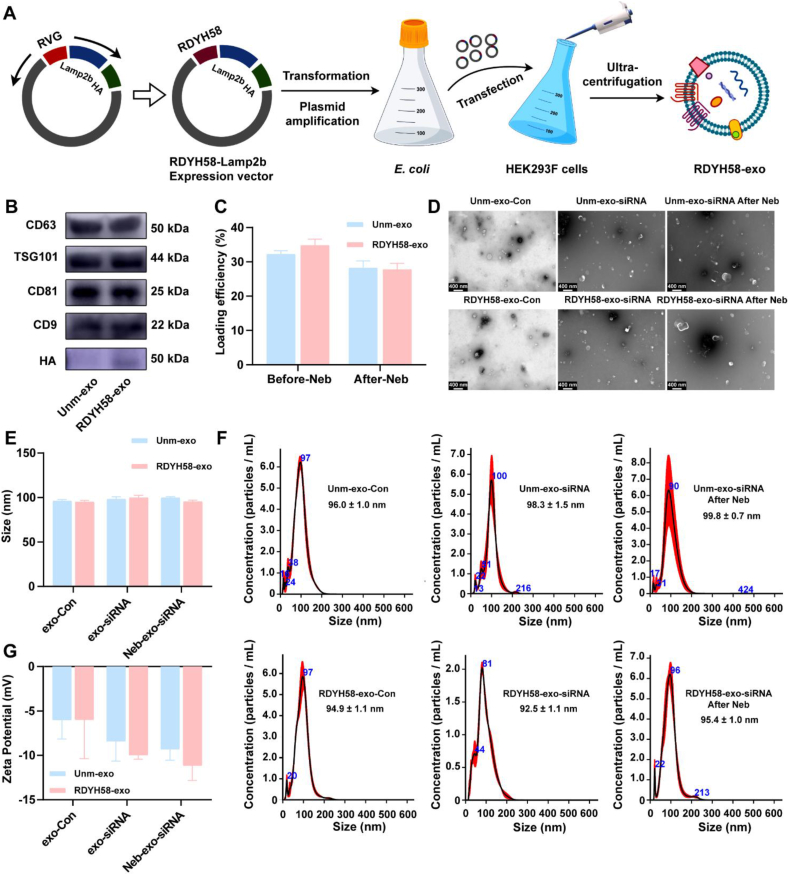
Characteristics of exosomes and exosomes loaded with siRNA before and after nebulization. (A) Schematic representation of the RDYH58-exo production process. (B) Positive markers (CD63, TSG101, CD81, and CD9), and an HA-tag of exosomes isolated from HEK293F cells using western blotting. (C) Loading efficiency of exosomes before and after nebulization. (D) Transmission electron microscopy images of exosomes. The bar is 400 nm. (E) Changes in the average particle size of exosomes. (F) Nanoparticle tracking analysis images of the size and concentration distribution of exosomes. (G) Changes in the zeta potential of exosomes.

A schematic representation of the RDYH58-exo production process is shown (Fig. 1A). The plasmid expressing RDYH58-Lamp2b-HA was transfected into HEK293F cells, and exosomes expressing RDYH58-Lamp2b-HA were isolated. The expression of RDYH58-Lamp2b-HA in exosomes was confirmed by Western blotting using an anti-HA antibody. HA-tagged RDYH58-exo showed positive results (Fig. 1B), whereas unmodified exosomes (unm-exo) did not express the HA-tagged protein. Both RDYH58-exo and unm-exo cells expressed the positive markers (CD63, TSG101, CD81, and CD9) (Fig. 1B). siFKBP10 was then loaded into RDYH58-exo and unm-exo cells using ultrasonic microfluidics. The loading efficiencies of RDYH58-exo and unm-exo before nebulization were 32.2% and 34.8%, and after nebulization decreased to 28.2% and 27.7% (Fig. 1C). TEM images revealed that the exosomes maintained their spherical shape (Fig. 1D). The size distribution of the exosomes was measured using NTA, and the particle sizes exhibited slight changes after drug loading and nebulization (Fig. 1E), ranging from 92.5 to 99.8 nm (Fig. 1F). Dynamic light scattering (DLS) measurements indicated a slight decrease in zeta potential after loading with siFKBP10 (Fig. 1G). The agarose gel electrophoresis results demonstrated that exosome-loaded siRNA remained detectable for up to 6 h, indicating that RDYH58-siFKBP10 exhibits significantly enhanced stability (Supporting Information Fig. S1).

### 3.2 Evaluation of BLM-induced fibroblast uptake of RDYH58-exo and unm-exo *in vitro*

**Figure 2:**
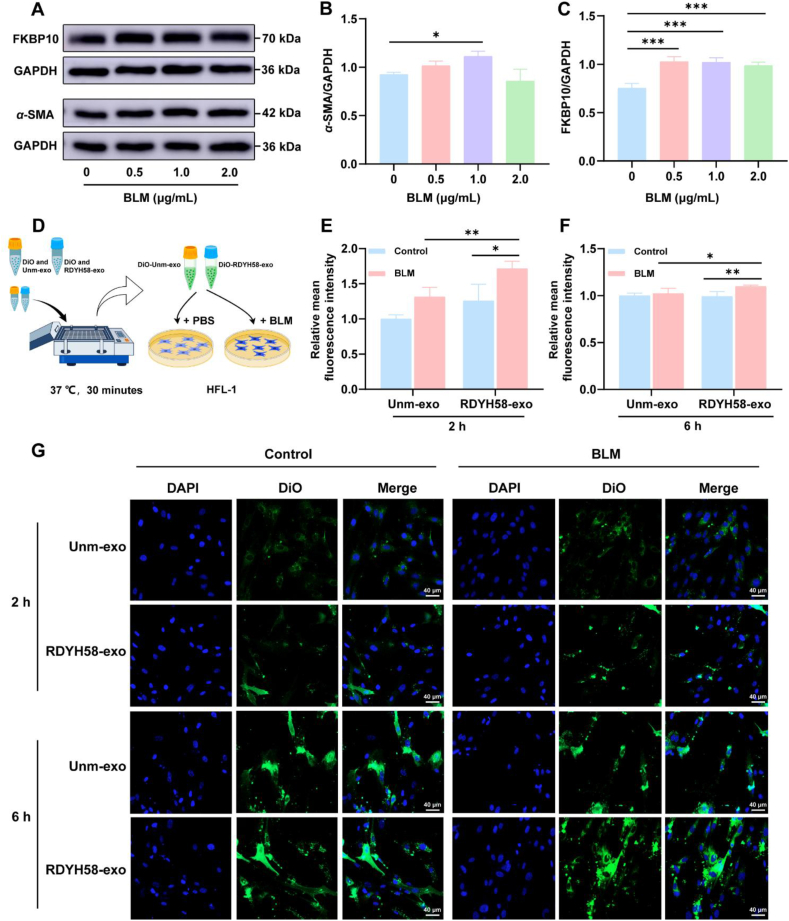
RDYH58-exo can be targeted and taken up by myofibroblasts *in vitro*. (A) Analysis of *α*-SMA and FKBP10 expression at the cellular level using Western blotting following induction with BLM. (B, C) Relative expression of *α*-SMA and FKBP10 levels was quantified (*n* = 4). (D) Schematic illustration of the assessment of DiO-labeled RDYH58-exo and unm-exo uptake levels in HFL-1 cells. (E, F) Analysis of the relative mean fluorescence intensity of cellular uptake of RDYH58-exo and unm-exo at 2 and 6 h (*n* = 3‒4). (G) Confocal laser microscopy images of cellular uptake of RDYH58-exo and unm-exo at 2 and 6 h by HFL-1 cells. Blue fluorescence indicates DAPI-stained nuclei, and green fluorescence indicates DiO-labeled exosomes (∗*P* < 0.05, *∗∗P* < 0.01, *∗∗∗P* < 0.001).

BLM can damage cells and induce the expression of the inflammatory factor TGF-*β*^30^, which subsequently leads to the phenotypic transformation of fibroblasts into myofibroblasts. *α*-SMA is a myofibroblast marker^31^. Fibroblasts were treated with different concentrations of BLM (0.5, 1.0, and 2.0 μg/mL) added to the cell culture medium and induced for a period of 48 h. Total protein isolated from the cells was subjected to Western blotting (Fig. 2A). *α*-SMA (Fig. 2B and Supporting Information Fig. S2) and FKBP10 (Fig. 2C) expression increased at a BLM concentration of 1.0 μg/mL, indicating the successful transformation of fibroblasts into myofibroblasts.

Exosomes, once released from cells, are readily taken up by other cells and naturally target damaged tissues and cells^32^. Physical and chemical damage to cells increases exosome uptake^33^. To validate the effectiveness of RDYH58 in targeting myofibroblasts, BLM-induced cellular damage was evaluated, and the cells were treated with RDYH58-exo and unm-exo for 2 and 6 h (Fig. 2D). RDYH58-exo was taken up more efficiently than unm-exo (Fig. 2E‒G). Thus, RDYH58-exo can effectively target myofibroblasts, which are considered the primary effector cells responsible for ECM production in pulmonary fibrosis^19^.

### 3.3 Targeting efficacy of RDYH58-exo in mice

**Figure 3:**
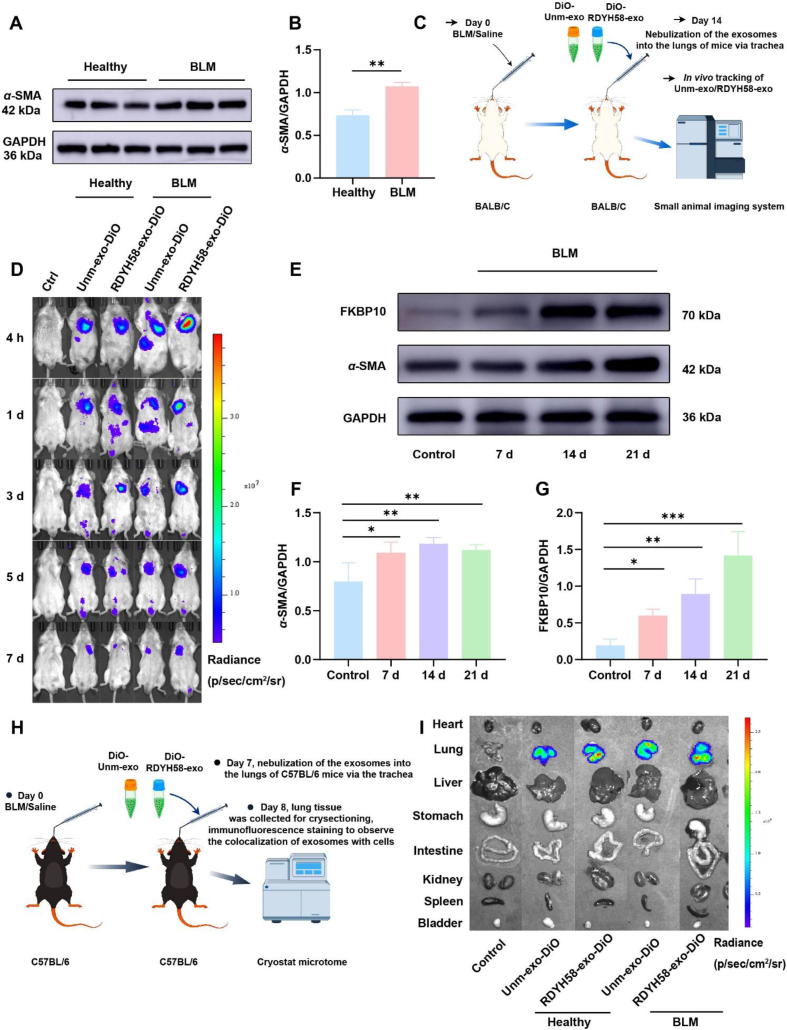
Biodistribution of DiO-labeled exosomes following nebulization. (A) *α*-SMA was separated by SDS-PAGE in the healthy controls and the BLM groups. (B) Relative expression level of *α*-SMA was quantified (*n* = 3). (C) Schematic illustration depicting the assessment of the *in vivo* distribution of DiO-labeled exosomes administered *via* aerosol inhalation in BALB/c mice after 14 days of exposure to BLM/PBS. (D) *In vivo* imaging images of small animals of DiO-labeled exosomes in BALB/c mice at 4 h and Days 1, 3, 5, and 7 post-nebulization. (E) Western blotting analysis shows the expression of *α*-SMA and FKBP10 in BLM-induced mice at 7, 14, and 21 days. (F, G) Relative expression levels of *α*-SMA and FKBP10 were quantified (*n* = 3‒4). (H) Schematic illustration depicting the assessment of tissue distribution following a one-day administration of DiO-labeled exosomes *via* aerosol inhalation in C57BL/6 mice after 7 days of exposure to BLM/PBS. (I) Tissue distribution of nebulized DiO-labeled exosomes in C57BL/6 mice at 1 day (∗*P* < 0.05, *∗∗P* < 0.01, *∗∗∗P* < 0.001).

To evaluate the ability of RDYH58-exo to specifically target myofibroblasts *in vivo*, a pulmonary fibrosis model of BALB/c (Fig. 3A and B) mice was established. The fluorescence signal in the pulmonary fibrotic BALB/c mice gradually weakened by day 3 after administration (Fig. 3C and D, and Supporting Information Fig. S3). Day 14 after the induction of pulmonary fibrosis in BALB/c mice using BLM, DiO-labeled exosomes were administered *via* intratracheal nebulization to pulmonary fibrotic mice and healthy controls, and exosome retention was observed^34^.

**Figure 4:**
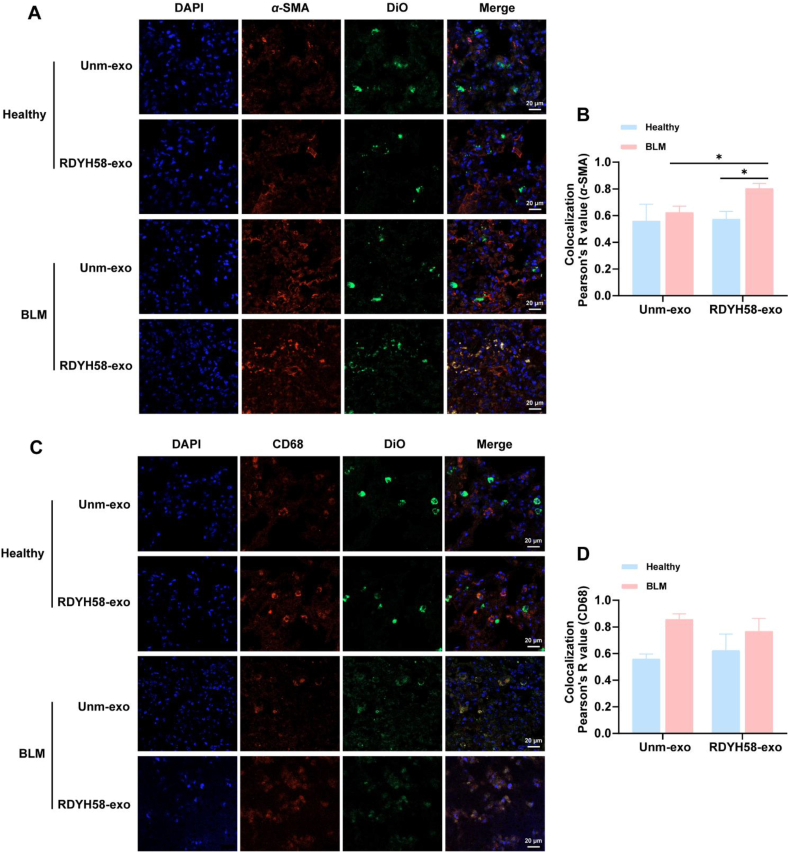
Immunofluorescence staining to observe the colocalization of RDYH58-exo and unm-exo with myofibroblasts, and macrophages. DiO-labeled exosomes were administered intratracheally to C57BL/6 mice, which reached the pulmonary cells. (A) Colocalization images of anti-*α*-SMA antibody for myofibroblasts and exosomes. (B, D) The Pearson’ R value indicates the degree of colocalization (*n* = 3‒5). (C) Colocalization images of anti-CD68 antibody for macrophages and exosomes (∗*P* < 0.05).

**Figure 5:**
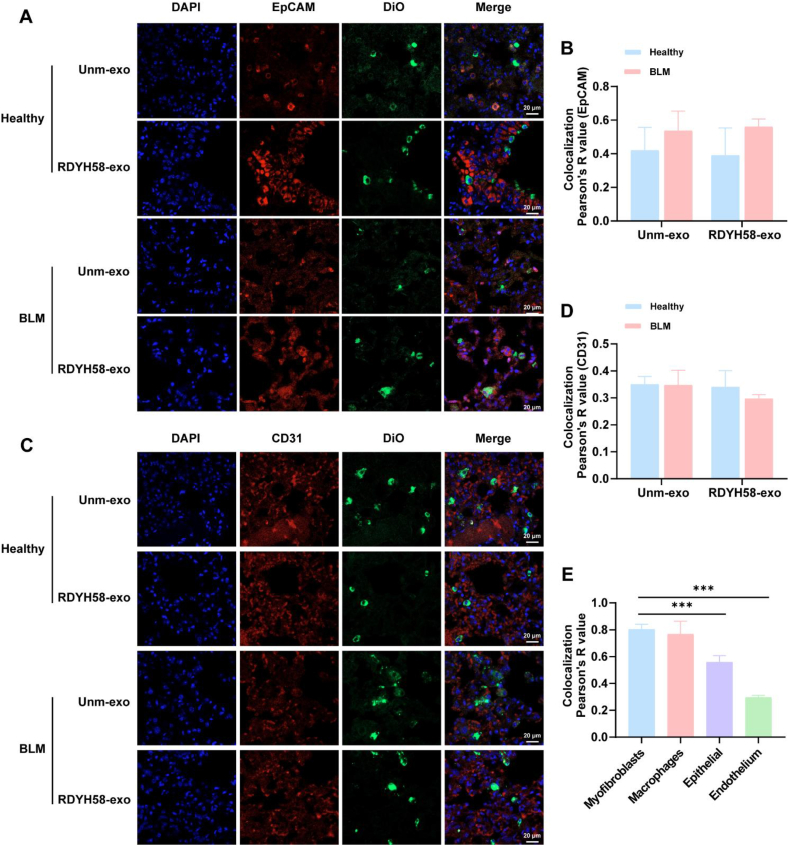
Immunofluorescence staining to observe the colocalization of RDYH58-exo and unm-exo with epithelial cells, and endothelial cells. (A) Colocalization images of anti-EpCAM antibody for epithelial cells and exosomes. (B, D) The Pearson’ R value indicates the degree of colocalization. (C) Colocalization images of anti-CD31 antibody for endothelium and exosomes. (E) The Pearson’ *R* value indicates the degree of colocalization between RDYH58-exo and myofibroblasts, macrophages, epithelial cells, and endothelial cells (*n* = 3‒5) (∗*∗∗P* < 0.001).

To further observe the types of cells targeted by RDYH58-exo and unm-exo in the lungs, a pulmonary fibrosis model of C57BL/6 (Fig. 3E‒G) mice was established. DiO-labeled exosomes were administered *via* intratracheal nebulization to both healthy controls and pulmonary fibrosis mice after Day 7 BLM exposure in C57BL/6 mice. Days 1 after administration, the organs were harvested and the fluorescence distribution was observed. The results revealed that the majority of the fluorescence signals were distributed in the lungs of the mice (Fig. 3H and I). The RDYH58-exo group exhibited significantly stronger fluorescence intensity (Supporting Information Fig. S4). Frozen sections of mouse lung tissue were prepared, and immunofluorescence staining was performed to observe the colocalization of RDYH58-exo and unm-exo with myofibroblasts, macrophages (Fig. 4), epithelial cells, and endothelial cells (Fig. 5A‒D). RDYH58-exo co-localized more with myofibroblasts compared with unm-exo. This was due to the higher expression of *α*-SMA in the BLM group. RDYH58-exo also showed strong fluorescence signals in macrophages, attributed to their phagocytic activity. In mice with pulmonary fibrosis, RDYH58-exo showed greater colocalization with myofibroblasts compared with epithelial and endothelial cells (Fig. 5E). This finding suggests a positive impact of the delivery of siFKBP10 to myofibroblasts for the treatment of pulmonary fibrosis.

### 3.4 RDYH58-siFKBP10 alleviated ECM protein deposition and inhibited migration of activated fibroblasts *in vitro*

**Figure 6:**
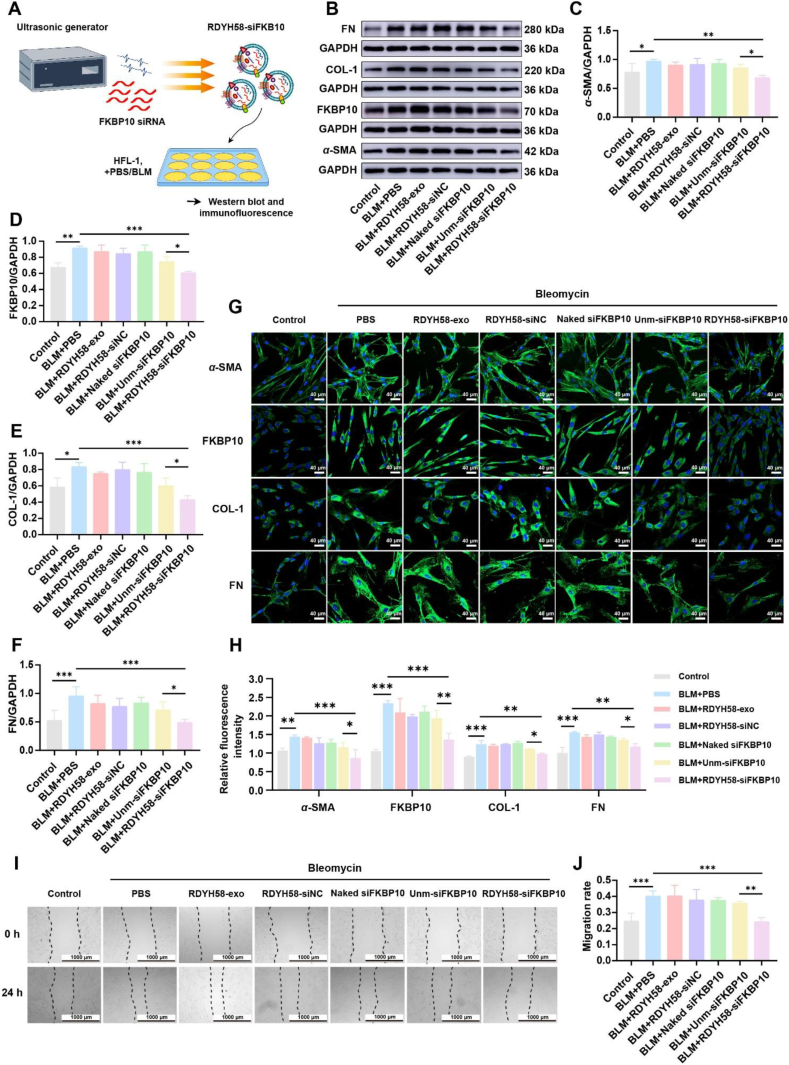
RDYH58-siFKBP10 inhibits fibroblast activation, reducing ECM deposition and cell migration. (A) Schematic illustration of the antifibrotic assessment of HFL-1 cells following BLM stimulation and treatment with RDYH58-siFKBP10. (B) Western blotting of *α*-SMA, FKBP10, COL-1, and FN in HFL-1 cells. (C‒F) Relative expression levels of *α*-SMA, FKBP10, COL-1, and FN were quantified (*n* = 3‒4). (G) Immunofluorescence staining of *α*-SMA, FKBP10, COL-1, and FN in HFL-1 cells (*n* = 3‒4). (H) Quantification of fluorescence intensity. (I) Migration of HFL-1 cells at 24 h. (J) Migration rate of migrated cells (*n* = 4; *∗P* < 0.05, *∗∗P* < 0.01, *∗∗∗P* < 0.001).

To evaluate the effects of RDYH58-siFKBP10 on BLM-induced cellular damage, the cells were treated with PBS, RDYH58-exo, RDYH58-exo loaded with control siRNA (RDYH58-siNC), naked siFKBP10, unm-siFKBP10, and RDYH58-siFKBP10 (Fig. 6A). After treatment with RDYH58-siFKBP10, the BLM-induced (1.0 μg/mL) upregulation of FKBP10 and ECM-related proteins was significantly reduced (Fig. 6B‒F). The decrease in *α*-SMA further indicated that RDYH58-siFKBP10 can inhibit fibroblast activation. The immunofluorescence results yielded the same conclusions (Fig. 6G and H). Wound healing assays showed that BLM-induced cell migration rate was reduced in the RDYH58-siFKBP10 treatment group (Fig. 6I and J).

### 3.5 Intratracheal nebulization of RDYH58-siFKBP10 in mice ameliorated BLM-induced lung inflammation

**Figure 7:**
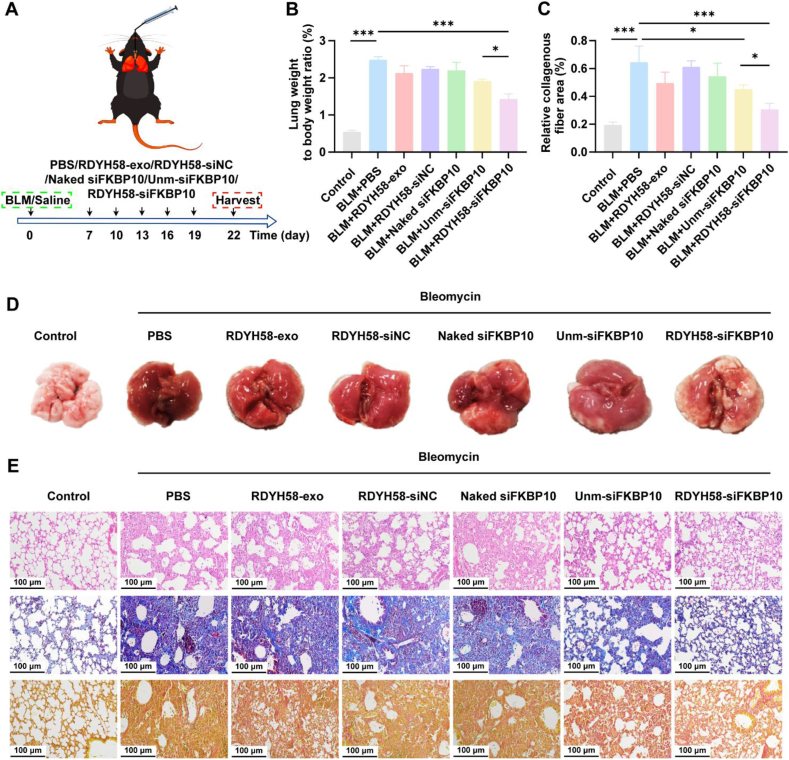
Delivery of siFKBP10 *via* nebulized RDYH58-exo ameliorates BLM-induced lung inflammation. (A) Flowchart illustrating the treatment experimental procedure for mice with pulmonary fibrosis. (B) Changes in lung weight of mice (*n* = 6). (C) Quantification of relative collagen area through Masson's trichrome (*n* = 3‒6). (D) Morphology of lung tissue in mice. (E) Histopathological analysis of H&E, Masson's trichrome, and Sirius Red staining (*∗P* < 0.05, *∗∗P* < 0.01, *∗∗∗P* < 0.001).

**Figure 8:**
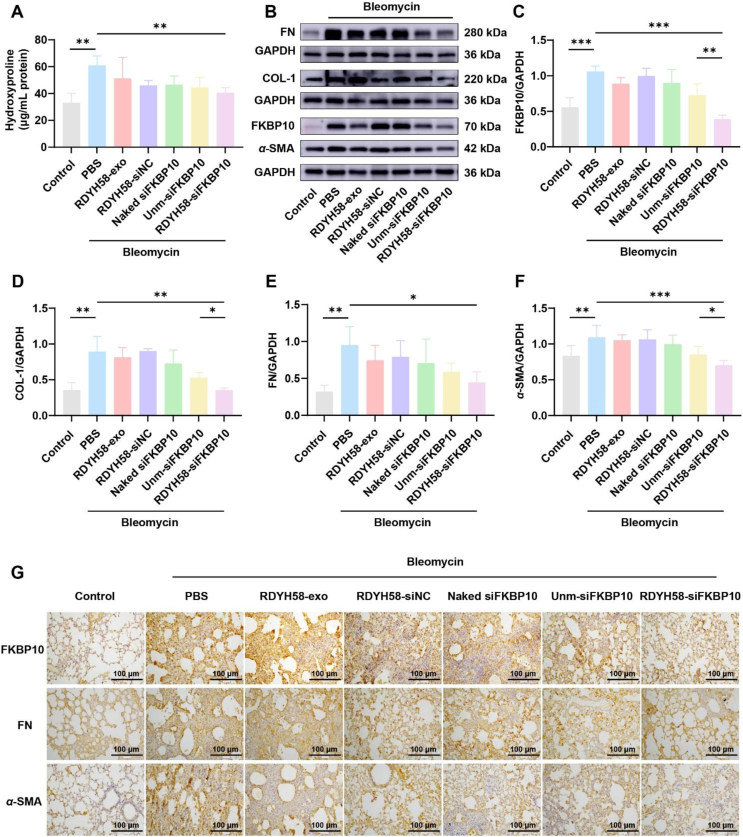
RDYH58-siFKBP10 alleviates BLM-induced pulmonary fibrosis in mice. (A) Detection of hydroxyproline content in mouse lung tissue (*n* = 3‒6). (B) Western blotting analysis of *α*-SMA, FKBP10, COL-1, and FN protein expression in lung tissues. (C‒F) Relative expression levels of FKBP10, COL-1, FN, and *α*-SMA were quantified (*n* = 3‒6). (G) Immunohistochemical staining of lung tissue sections (∗*P* < 0.05, *∗∗P* < 0.01, *∗∗∗P* < 0.001).

To detect the expression of FKBP10 in the lungs of C57BL/6 mice with pulmonary fibrosis^35^, tissues were collected on Days 0, 7, 14, and 21, and proteins were extracted for Western blot analysis (Fig. 3E). The results showed that the expression of FKBP10 and *α*-SMA was upregulated on Day 7 of BLM induction (Fig. 3F and G). Day 7 after BLM exposure, treatments with PBS, RDYH58-exo, RDYH58-siNC, naked siFKBP10, unm-siFKBP10, or RDYH58-siFKBP10 were administered to slow the progression of pulmonary fibrosis. The dosing frequency was every 3 days and 1.5 mg/kg of siFKBP10, and changes in mouse body weight were recorded throughout the treatment period (Fig. 7A). Treatment with RDYH58-siFKBP10 mitigated local congestion and necrosis in the fibrotic lungs and reduced lung weight, histological analysis using H&E, Masson's trichrome, and Sirius Red staining demonstrated that RDYH58-siFKBP10 effectively ameliorated the extensive fibrotic tissue proliferation, thickened alveolar septa, and other structural changes induced by BLM (Fig. 7B‒E). Further evaluation of collagen secretion levels throughout the lungs by detected hydroxyproline expression^36^ showed that RDYH58-siFKBP10 significantly downregulated hydroxyproline content in the lungs compared to that in the BLM group (Fig. 8A), effectively inhibiting collagen accumulation. TGF-*β*1 serves as a key biomarker for fibrotic fibroblasts^37^. Western blot analysis demonstrated that RDYH58-siFKBP10 treatment significantly reduced TGF-*β*1 expression levels (Supporting Information Fig. S5).

### 3.6 RDYH58-siFKBP10 exerts an antifibrotic effect by inhibiting the expression of fibrotic factors in the lungs

The development of IPF is accompanied by the release of inflammatory factors such as TGF-*β*1 and connective tissue growth factor, leading to the activation and proliferation of lung fibroblasts. These fibroblasts subsequently differentiate into myofibroblasts that overexpress *α*-SMA and secrete excessive amounts of fibrotic factors, resulting in excessive deposition of the ECM^23^^,^^38^. FKBP10 expression was upregulated in fibrotic lungs (Fig. 3E and G) and was localized in myofibroblasts and macrophages^25^. After administration of RDYH58-siFKBP10 to BLM-exposed pulmonary fibrosis mice, Western blotting results showed a reduction in FKBP10 expression (Fig. 8B and C), along with a decrease in ECM-related proteins, including COL-1, FN, and *α*-SMA (Fig. 8D‒F). Immunohistochemistry results demonstrated that the administration of RDYH58-siFKBP10 reduced the expression of FN and *α*-SMA in the excessive ECM (Fig. 8G), while other treatment groups did not exhibit the same therapeutic effects. siFKBP10 exhibited potent antifibrotic potential by inhibiting collagen secretion.

### 3.7 RNA-seq results revealed that RDYH58-siFKBP10 inhibits cell differentiation and migration in the IPF mouse model

**Figure 9:**
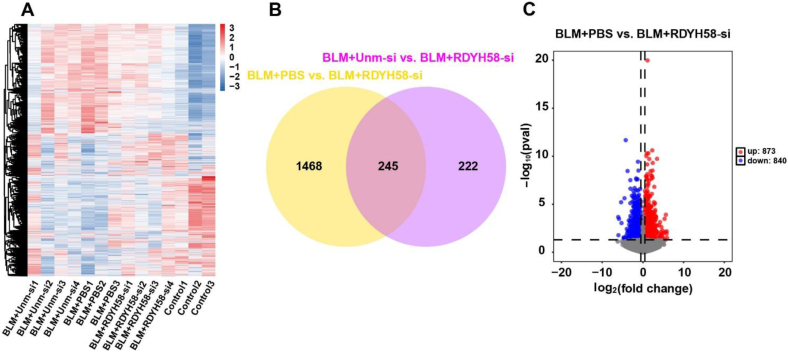
Differential gene expression in the control, BLM + PBS, BLM + unm-siFKBP10, and BLM + RDYH58-siFKBP10-treated mice. (A) Heatmap showing the upregulated (red) and downregulated (blue) genes among the control, BLM + PBS, BLM + unm-siFKBP10, and BLM + RDYH58-siFKBP10 groups. (B) Venn diagram of differentially expressed genes in BLM + PBS *vs.* BLM + RDYH58-siFKBP10 and BLM + unm-siFKBP10 *vs.* BLM + RDYH58-siFKBP10 groups. (C) Scattergram showing the fold change (FC) expression of genes between the BLM + PBS and BLM + RDYH58-siFKBP10 groups.

To evaluate the effects of RDYH58-siFKBP10 treatment on gene expression, transcriptome sequencing was performed in the control, BLM + PBS, BLM + unm-siFKBP10, and BLM + RDYH58-siFKBP10 groups after treatment completion. Heatmaps were generated to visualize the changes in gene expression across the different groups (Fig. 9A). Sequencing reads were aligned to genomic regions or exons, and gene expression levels were quantified using two normalization methods: Fragments per kilobase of exon model per million mapped fragments (FPKM) and Transcripts Per Million (TPM). The String Tie tool was used to quantify gene expression. Differential expression analysis was conducted using DESeq2, with a log_2_ (fold change) > 0.5 and *P* value < 0.05 set as the criteria for selecting differentially expressed genes. Compared to the BLM + PBS group, BLM + RDYH58-siFKBP10 treatment resulted in the differential expression of 1713 genes. In the BLM + RDYH58-siFKBP10 group, 873 genes were upregulated and 840 were downregulated (Fig. 9B and C). The selected differentially expressed genes included several genes related to ECM and cell migration, including FKBP10, matrix metalloproteinase 14, FN1, and COL-1 (Supporting Information Table S1). Compared to the BLM + unm-siFKBP10 treatment group, the BLM + RDYH58-siFKBP10 group showed differential expression of 467 genes, with 245 genes being shared as differentially expressed when compared to the BLM + PBS group. Kyoto Encyclopedia of Genes and Genomes (KEGG) enrichment analysis revealed that, compared to the BLM + PBS group, the BLM + RDYH58-siFKBP10 group showed upregulation or downregulation of genes involved in pathways such as ECM-receptor interactions. The top 20 enriched pathways demonstrated that compared to the BLM + PBS group, the BLM + RDYH58-siFKBP10 group was significantly enriched in various pathways related to cell migration and ECM, including the Wnt signaling pathway, PI3K-Akt signaling pathway, and ECM-receptor interaction. Gene Ontology (GO) enrichment analysis of differentially expressed genes related to biological processes, cellular components, and molecular functions showed that the top 20 GO enrichments indicated that the BLM + RDYH58-siFKBP10 treatment group positively regulated angiogenesis and ECM organization (Supporting Information Fig. S6; Table S2).

## 4 Discussion

In recent years, exosomes have emerged as promising therapeutics for lung diseases. Many exosome-based approaches have been developed for treating IPF, including engineered exosomes for the delivery of bioactive substances^39^^,^^40^. However, the mechanisms underlying IPF are complex and involve multiple cell types and molecular pathways, posing significant challenges to its prevention, diagnosis, and treatment^16^. Current therapeutic agents such as pirfenidone and nintedanib can only temporarily delay the progression of IPF, demonstrate no significant effects on pulmonary inflammation, persistent fibrosis, or improvement in lung function^41^^,^^42^. These treatments also face challenges including limited efficacy and adverse effects such as hepatotoxicity and gastrointestinal complications. Exosomes possess low immunogenicity and exceptional ability to penetrate biological barriers^38^^,^^43^, representing a highly promising direction for future research and therapeutic development. In this study, we report the antifibrotic effects of engineered exosomes loaded with siFKBP10 on BLM-induced cellular damage and in animal models of pulmonary fibrosis. RDYH58-siFKBP10 was delivered to myofibroblasts *via* nebulization, which inhibited collagen expression, cell differentiation, and migration, thereby reducing ECM deposition and alleviating fibrosis. Additionally, RNA-seq analysis revealed that the delivery of RDYH58-siFKBP10 positively regulated pathways related to cell migration, including the PI3K-Akt signaling pathway and ECM-receptor interaction. To validate these findings, we examined the protein expression of MMP9, a key downstream effector molecule in the PI3K–Akt pathway. The results demonstrated that RDYH58-siFKBP10 significantly suppressed MMP9 expression levels (Supporting Information Fig. S7)^44^.

Exosomes were isolated from HEK293F cells and loaded with siFKBP10. The loading efficiencies of unm-exo and RDYH58-exo were 32.2% and 34.8%, respectively. HEK293F cells have been thoroughly characterized and demonstrate excellent preclinical safety in both *in vitro* and *in vivo* tests^38^. The exosomes extracted from HEK293F cells after scale-up from shake flasks to stirred-tank bioreactor cultures maintained high yield and particle integrity^38^^,^^45^, which would facilitate their therapeutic development. However, the loading efficiency of drug-loaded exosomes remains a limiting factor for their application. Current reports indicate that electroporation can achieve a loading efficiency of approximately 30%^2^. However, this method can cause membrane aggregation^46^ and siRNA precipitation^47^, which are critical issues because the integrity of the membrane and drug is crucial for determining the efficiency of loading and drug delivery. In contrast, the sonication method is relatively mild, but can also cause an increase in exosome size^10^. Ultrasonic microfluidics have been used to prepare stable and uniform liposomes^48^. When used for the loading of siRNA into exosomes, this method has a minimal impact on the structure and size of exosomes, which can be attributed to the uniform ultrasound distribution and excellent mixing performance of the ultrasonic microreactor.

Exosomes are aerosolized and delivered to the lungs through the trachea, resulting in widespread distribution throughout the lungs. Their small particle size (<1 μm in diameter) enables them to easily reach the alveoli, allowing for efficient deposition of the aerosolized exosomes to the lungs^49^. This administration method, being more direct, not only avoids the clearance of the drug by multiple layers of macrophages but has also been validated for its feasibility and safety^34^^,^^50^ (Supporting Information Fig. S8). *In vivo* imaging demonstrated that aerosolized exosomes exhibited extensive distribution in the lungs, enhancing their bioavailability in the lung tissue. *Ex vivo* tissue analysis indicated that the majority of exosomes were predominantly distributed in the lungs 1-day post-administration. This process minimizes side effects in off-target sites while improving therapeutic efficacy in the targeted area. The inhalation of exosomes derived from HEK293T cells is known to result in their high accumulation exclusively within the target organ, the lungs^51^. Shi et al.^34^ delivered mesenchymal stem cell-derived exosomes to the lungs *via* intratracheal nebulization. The strongest fluorescence intensity at 24 h appeared in the lung, starting from 1 h post-nebulization, and the fluorescence intensity in the lung gradually decreased up to 28 days.

Notably, the complex microenvironment of disease sites limits the ability of natural exosomes to reach specific tissues and cells^52^. Exosomes expressing these fusion proteins can be isolated by constructing plasmids that express the fusion proteins. Compared with unm-exo, RDYH58-exo successfully targeted myofibroblasts. Exosomes are derived from a wide range of sources. However, exosomes produced by cells inherit the homing ability of their parent cells^53^, which limits the choice of cell sources. Numerous exosome engineering strategies have been developed to enhance their targeting to specific cells. Advanced glycation end-product receptor (RAGE) is an important factor in acute lung injury (ALI) and represents a potential therapeutic target for ALI. Researchers have leveraged the binding interaction between RAGE and RAGE-binding peptides (RBP) to design a cDNA construct that expresses a fusion protein of RBP and the exosomal membrane protein lamp2b. When this cDNA was transfected into cells, exosomes capable of targeting lung injury sites were isolated^39^. By developing a mixed drug delivery system comprising clodronate (CLD)-loaded liposomes and fibroblast-derived exosomes (EL-CLD), the exosomes were endowed with two characteristics: non-specific phagocytosis inhibition and fibroblast homing. This system was designed to treat pulmonary fibrosis^54^. In this study, we constructed cDNA expressing RDYH58-lamp2b-HA, transfected it into HEK293F cells, and isolated exosomes expressing the RDYH58-lamp2b-HA fusion protein. *In vitro* experiments demonstrated that in both untreated cells and BLM-treated fibroblasts, RDYH58-exo was taken up more efficiently by damaged cells at 2 and 6 h post-treatment. To rule out the possibility that exosomes themselves have an inherent tropism for damaged cells, we compared the uptake of RDYH58-exo and unm-exo in BLM-treated fibroblasts at 2 and 6 h. RDYH58-exo exhibited superior ability to target myofibroblasts compared to unm-exo. To evaluate the targeting ability of RDYH58-exo *in vivo*, a mouse model of IPF was established. DiO-labeled exosomes were delivered to both healthy and BLM-treated mice. After 24 h, lung tissue cryosections were prepared and subjected to immunofluorescence staining. Compared with the epithelial and endothelial cells, RDYH58-exo exhibited greater colocalization with myofibroblasts. In addition to myofibroblasts, RDYH58-exo showed significant colocalization with macrophages in IPF mice. Related studies demonstrated that neutrophil-derived exosomes *in vivo* are predominantly engulfed by macrophages, indicating phagocytes as their primary target cells^55^. This could be attributed to the fact that macrophages, which are important phagocytic cells, play a crucial role in the clearance of foreign substances.

Administration of RDYH58-siFKBP10 *via* nebulization in the lungs of IPF model mice alleviated symptoms of fibrosis. FKBP10 is currently recognized as a therapeutic target for IPF. The use of BLM to induce cellular damage results in the upregulation of FKBP10. Following treatment with RDYH58-siFKBP10, FKBP10 expression was downregulated, leading to a reduction in the expression of ECM-related proteins and inhibition of the activation and migration of lung fibroblasts. As previously reported, siFKBP10 regulates fibroblast migration by modulating collagen^56^ and *α*-SMA synthesis^57^. Notably, in mouse models, Day 7 after BLM induction, a significant difference in the expression of FKBP10 and *α*-SMA was noted compared to the control group, with FKBP10 expression primarily localized in myofibroblasts and macrophages. The upregulation of *α*-SMA expression is characteristic of IPF. Furthermore, FKBP10 expression gradually increased on Days 14 and 21 after BLM exposure. To inhibit the development of fibrosis, we administered RDYH58-siFKBP10 *via* tracheal spray for a two-week therapeutic regimen to the lungs of mice on Day 7 after BLM exposure. Compared with other treatment groups, the BLM + RDYH58-siFKBP10 group demonstrated a reduction in lung collagen expression and lung weight, which was achieved by suppressing the overexpression of FKBP10. Liang et al.^58^ used an FKBP10 siRNA to inhibit fibroblast activity and ECM deposition, attenuating hypertrophic scar formation. This confirms the critical role of siFKBP10 in inhibiting collagen synthesis during IPF treatment. This targeted delivery system may possess therapeutic advantages over currently available clinical drugs (Supporting Information Fig. S9). Studies have demonstrated that fibroblast migration and aberrant angiogenesis collectively drive the progression of pulmonary fibrosis. Specifically, pro-fibrotic cytokines such as TGF-*β*1 activate fibroblasts, promoting their differentiation into myofibroblasts that migrate to the alveolar interstitium. Meanwhile, injury-induced dysfunction of parenchymal cells leads to dysregulated vascular endothelial activity and pathological angiogenesis. These mechanisms synergistically exacerbate tissue fibrosis^59^, ^60^, ^61^. RNA-seq analysis indicated that RDYH58-siFKBP10 positively regulated factors related to cell migration and ECM formation. These findings provide possibilities for identifying new therapeutic targets and exploring the mechanisms associated with IPF treatment using siFKBP10. In summary, RDYH58-siFKBP10 demonstrated potent anti-pulmonary fibrotic effects.

Although the results are encouraging, this study had some limitations. This study focused on short-term effects, and there remains limited understanding of the long-term effects of RDYH58-siFKBP10 use, including its safety and the sustainability of its efficacy. Although the effect of RDYH58-siFKBP10 on the expression of certain fibrotic proteins has been analyzed, further experiments are required to deepen our understanding of its specific mechanisms of action, particularly the precise regulation of specific biological processes. This study confirms that exosomes can reach lung cells; however, a critical limitation remains in evaluating the pulmonary delivery efficiency of nebulized exosomes: the lack of aerodynamic characterization during the nebulization process hinders accurate assessment of the actual proportion of exosomes reaching the lungs and their subsequent drug delivery efficiency. Addressing these limitations is crucial for advancing our understanding and the potential clinical application of RDYH58-siFKBP10 in the treatment of IPF.

## 5 Conclusions

In conclusion, this study utilized exosomes targeting myofibroblasts as carriers, loaded with siFKBP10 using ultrasonic microfluidics to form the RDYH58-siFKBP10 combination system, and investigated its effects on pulmonary fibrosis. *In vitro* and *in vivo* studies demonstrated that RDYH58-siFKBP10 successfully targets myofibroblasts and alleviates IPF by inhibiting the migration and activation of fibroblasts as well as downregulating the expression of ECM proteins, thereby improving mouse survival. RNA-seq analysis further validated and revealed the possible molecular mechanisms. Thus, FKBP10 is an important therapeutic target for the treatment of pulmonary fibrosis in myofibroblasts. This study provides a novel therapeutic strategy for the treatment of IPF.

## Author contributions

Ranran Yuan, Zhen Mu, Houqian Zhang, Yu Tian, Quanlin Xin, Qingchao Tu, Yan Zhang, Yanqiu Li, Zhiwen Zhang, Yongchao Chu, Aiping Wang, Jingwei Tian, Hongbo Wang, Chong Qiu, and Yanan Shi completed Conceptualization, Formal analysis, Data curation, and Methodology; Ranran Yuan, Zhen Mu, Hongbo Wang, Chong Qiu, and Yanan Shi completed the Writing-original draft; Yanan Shi, Chong Qiu, Hongbo Wang, and Ranran Yuan completed the Writing-review & editing.

## Conflicts of interest

The authors have no conflicts of interest to declare.

## Appendix A Supporting information

The following is the Supporting Information to this article.
