# Supplementary material for: RDYH58 functional exosomes targeting myofibroblasts loaded with siFKBP10 for inhibition of collagen biosynthesis and secretion of IPF
Source: Acta Pharm Sin B. 2025 Aug 27;15(12):6681–97. doi: 10.1016/j.apsb.2025.08.017 (PMC12750174; doi:10.1016/j.apsb.2025.08.017)
Supplement: Multimedia component 1 [file mmc1.pdf]

Supporting Information for

ORIGINAL ARTICLE

RDYH58 functional exosomes targeting myofibroblasts loaded with siFKBP10 for inhibition of collagen biosynthesis and secretion of IPF

Ranran Yuan[a,†], Zhen Mu[a,†], Houqian Zhang[a], Yu Tian[a], Quanlin Xin[a], Qingchao Tu[b], Yan Zhang[a], Yanqiu Li[c], Zhiwen Zhang[d], Yongchao Chu[a], Aiping Wang[a], Jingwei Tian[a], Hongbo Wang[a,*], Chong Qiu[b,*], Yanan Shi[a,*]

[a]*School of Pharmacy, Key Laboratory of Molecular Pharmacology and Drug Evaluation, Ministry of Education, Collaborative Innovation Center of Advanced Drug Delivery System and Biotech Drugs in Universities of Shandong, Yantai University, Yantai 264005, China*

[b]*State Key Laboratory for Quality Ensurance and Sustainable Use of Dao-di Herbs, Artemisinin Research Center, and Institute of Chinese Materia Medica, China Academy of Chinese Medical Sciences, Beijing 100700, China*

[c]*Department of Respiratory and Critical Care Medicine, Yantai Yuhuangding Hospital, Affiliated with the Medical College of Qingdao, Yantai 264200, China*

[d]*School of Pharmacy, Fudan University, Shanghai 200437, China*

Received 20 February 2025; received in revised form 3 June 2025; accepted 20 July 2025

*Corresponding authors.

E-mail addresses: shiyanan@ytu.edu.cn (Yanan Shi), cqiu@icmm.ac.cn (Chong Qiu), wanghongbo@ytu.edu.cn (Hongbo Wang).

[†]These authors made equal contributions to this work.

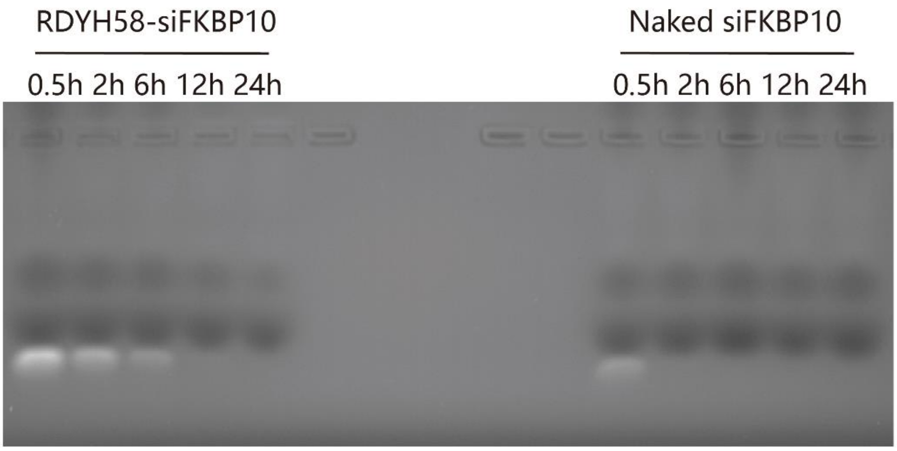

Figure S1 Stability of naked siFKBP10 and RDYH58-siFKBP10 after treatment with RNase at different times.

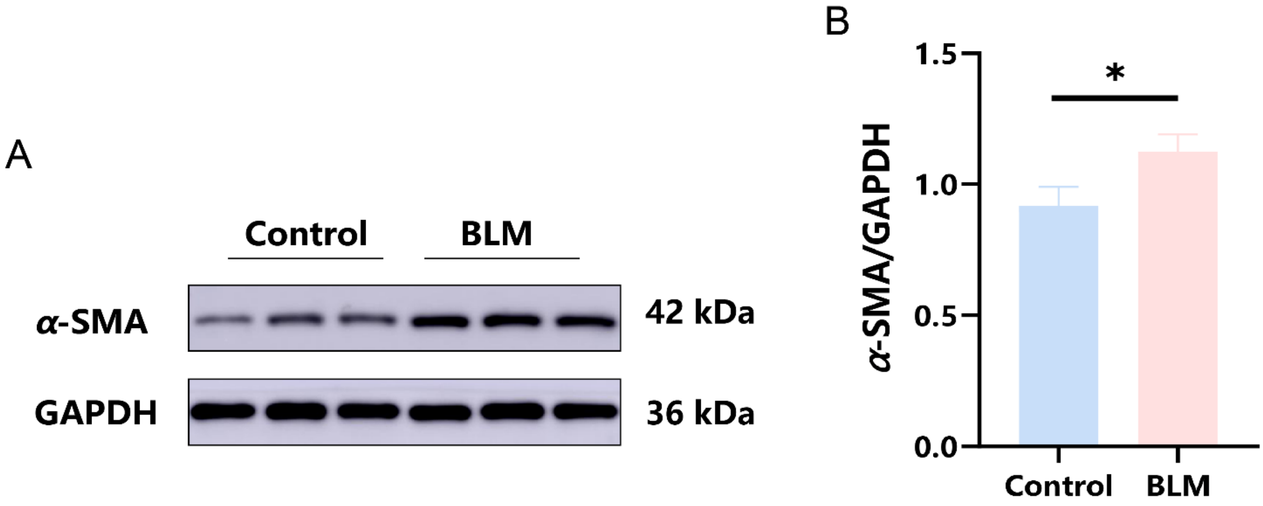

Figure S2 (A) Analysis of α-SMA expression at the cellular level using Western blotting following induction with BLM (1 μg/mL). (B) Relative expression of α-SMA level was quantified (n=3) (*P < 0.05).

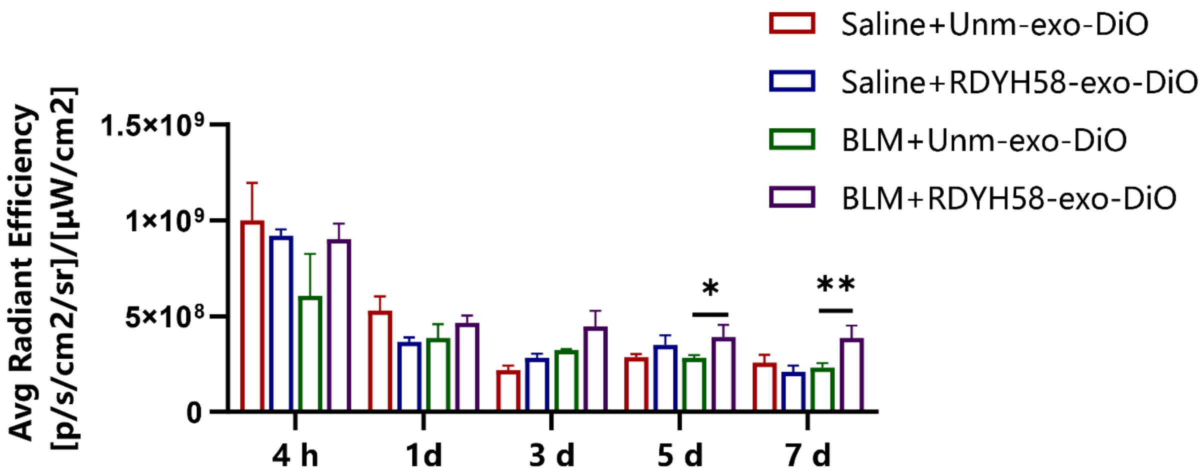

Figure S3 Semi-quantitative analysis of fluorescence intensity from DiO-labeled exosomes in BALB/c mice *via in vivo* imaging at 4 h, and Days 1, 3, 5, and 7 post-nebulization (*n*=3–4) (*$P < 0.05$, **$P < 0.01$).

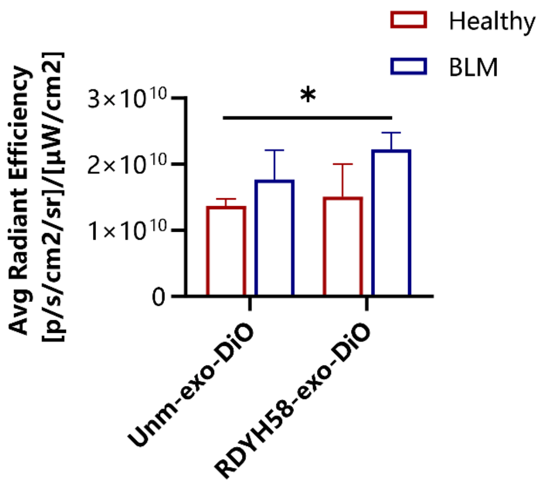

Figure S4 Semi-quantitative analysis of tissue distribution following nebulized administration of DiO-labeled exosomes in C57BL/6 mice at 24 h post-treatment (*n*=3) (*$P < 0.05$).

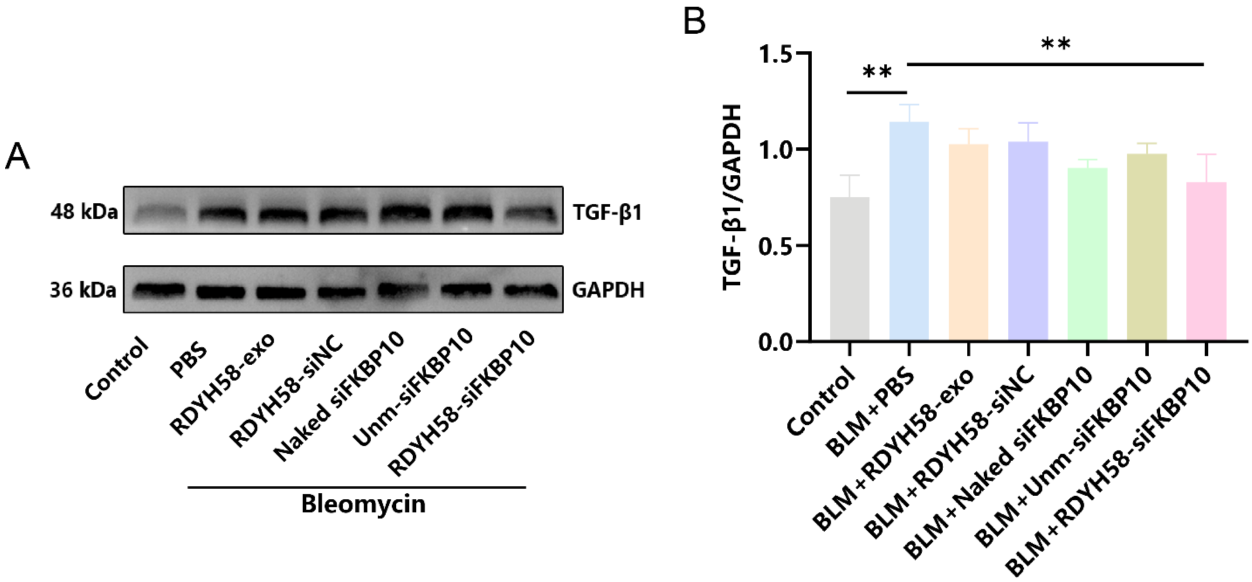

Figure S5 RDYH58-siFKBP10 significantly suppresses the expression of the fibrotic factor TGF-*β*1. (A) Western blotting analysis of TGF-*β*1 protein expression in lung tissues. (B) Relative expression level of TGF-*β*1 is quantified (*n*=3–6) (***P* < 0.01).

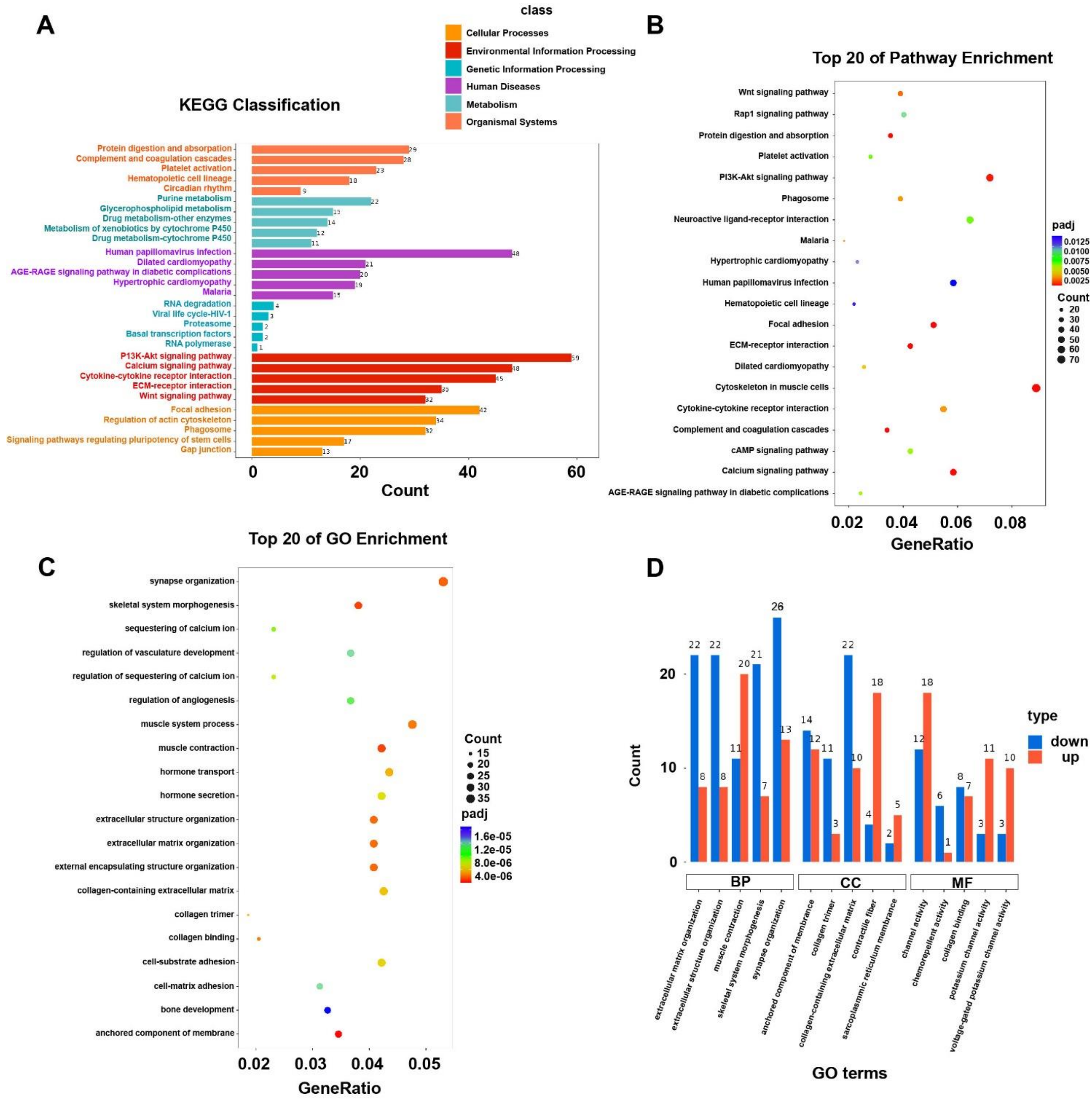

Figure S6 (A) KEGG classification of the differential genes in BLM + PBS compared with the BLM + RDYH58-siFKBP10 groups. (B) Top 20 enriched pathways for the differentially expressed genes, including the PI3K–AKT signaling pathway and the cytoskeleton in muscle cells related to cell migration. (C) Top 20 GO enrichment terms showing the participation of differential genes with collagen-containing extracellular matrix. (D) GO classification for the differential genes in BLM+PBS compared with BLM+RDYH58-siFKBP10 groups.

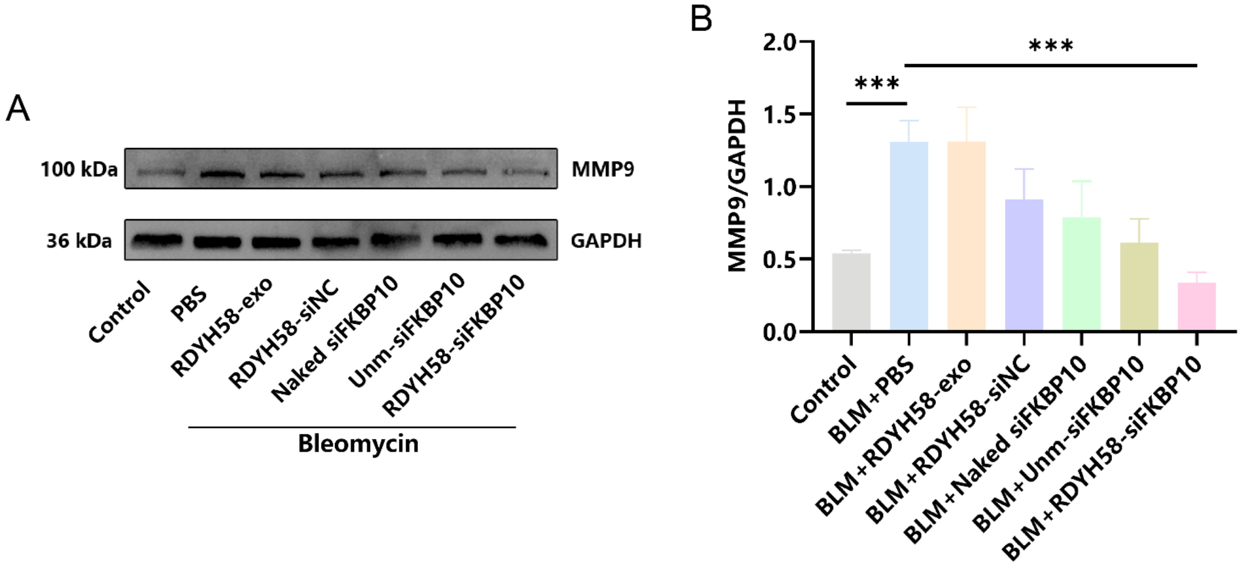

Figure S7 RDYH58-siFKBP10 regulates the fibrotic process *via* the PI3K–Akt downstream protein MMP9 (A) Western blotting analysis of MMP9 protein expression in lung tissues. (B) Relative expression level of MMP9 is quantified (n=3–6) (***P < 0.001).

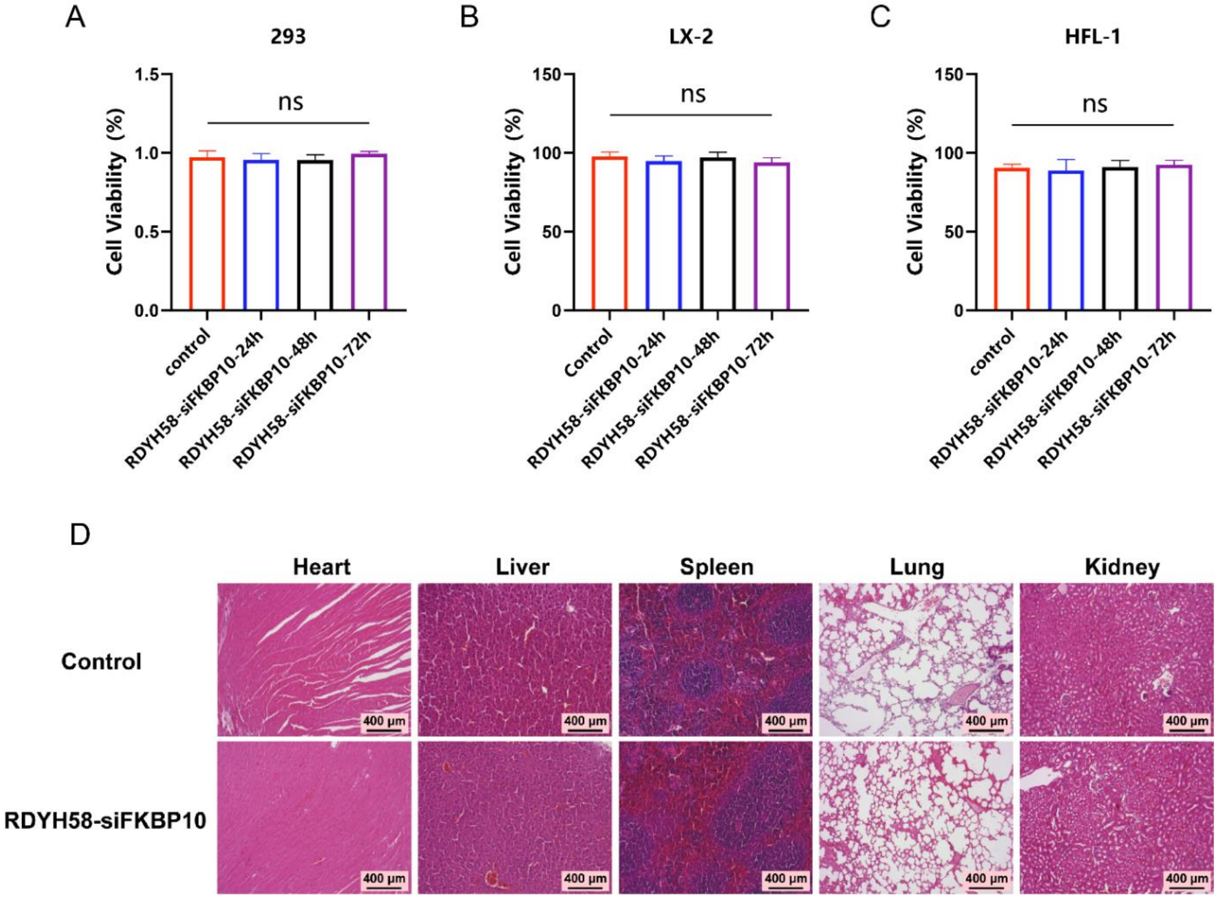

Figure S8 RDYH58-siFKBP10 demonstrates a favorable safety profile. Cytotoxicity assays of RDYH58-siFKBP10 treated for 24, 48, and 72 h in (A) Human embryonic kidney cells (HEK 293),

(B) Human hepatic stellate cells (LX-2), (C) Human lung fibroblasts (HFL-1) (n=4). (D) H&E staining of the heart, liver, spleen, lung, and kidney in mice 21 days after pulmonary nebulization of RDYH58-siFKBP10. ($^{ns}P > 0.05$).

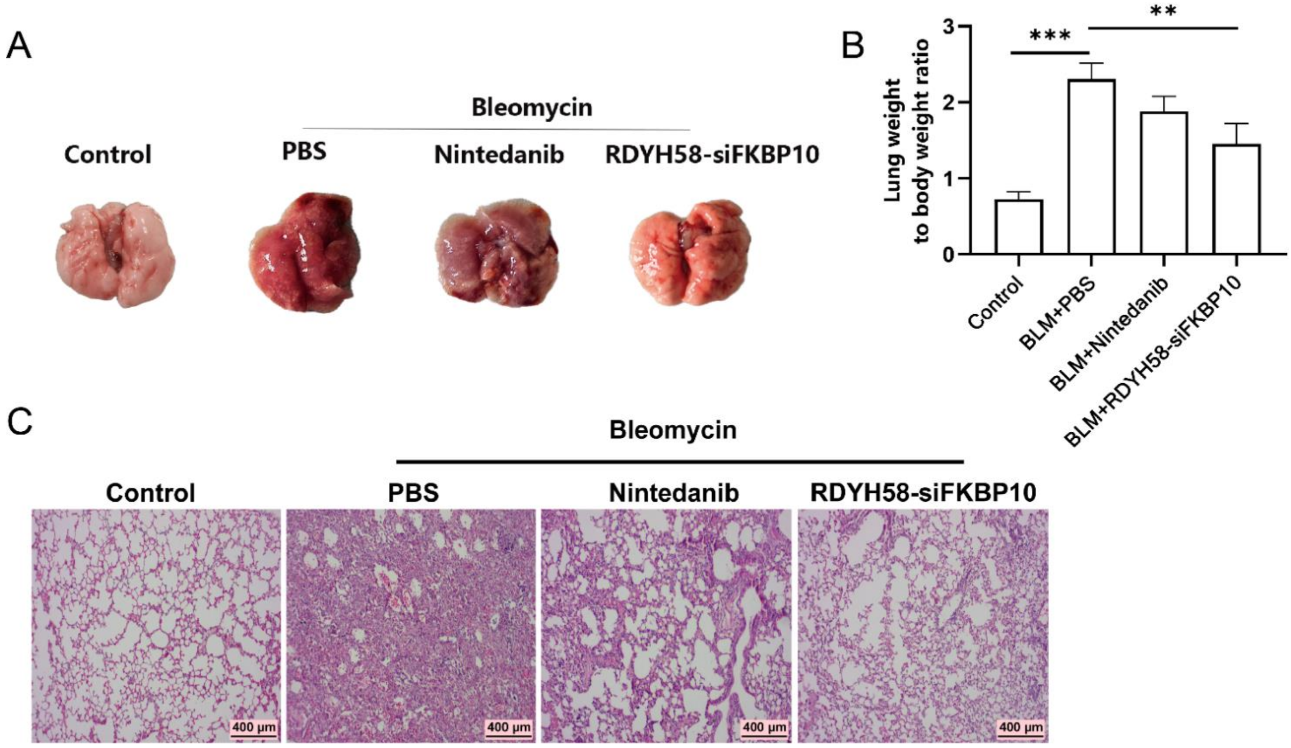

Figure S9 (A) Morphology of lung tissue in mice. (B) Changes in lung weight of mice (n=3). (C) H&E staining of lung tissue sections from mice treated with RDYH58-siFKBP10 and nintedanib (**P < 0.01, ***P < 0.001).

Table S1 Representative differentially expressed genes related to cell migration and ECM in the comparison between BLM and BLM+RDYH58-siFKBP10 groups.

Gene ID	Gene name	Description	log_2 FC	*P* value
ENSMUSG00000026193	FN1	Fibronectin 1	–1.948	0.000002261
ENSMUSG00000000957	MMP14	Matrix metallopeptidase 14	–0.9685	0.002351
ENSMUSG00000001506	COL-1	Collagen, type I	–1.489	0.002418
ENSMUSG00000001555	FKBP10	FK506 binding protein 10	–1.104	0.001432

Table S2 Representative differentially expressed genes related to cell migration and ECM in the comparison between BLM and BLM+RDYH58-siFKBP10 groups.

Category	ID	*P* value	Gene ID
Biological Processes	GO:0030198	1.16091205598647E-09	Crtap/Tnxb/Qsox1/Loxl2/Dpp4/Abi3bp/Csgalnact1/Lum/Adamts2/Olfml2b/Col16a1/Sh3pxd2b/Npnt/Ecm2/Adamts3/Zfp469/Adamts6/Flrt2/Adamts12/Adamts16/Mmp12/Mmp13/Dspp/Colq/Adamts17/Adamts14/Ext1/Col23a1/Fbln2/Col28a1
Biological Processes	GO:1901342	3.7120825698134E-08	Itgb2l/Tgfbr2/Cd59a/Lif/Tbxa2r/Ccl2/Fgf1/Fgf2/Serpine1/Vash2/Ppp1r16b/Thbs1/C3ar1/Prkd2/Il17f/Aplnr/Cxcr4/Ccbe1/C5ar1/Adam12/Klf2/Hmga2/Lep/Sphk1/Ghrl/Chil1/Hhip
